# The histological characteristics and clinical outcomes of lung cancer in patients with combined pulmonary fibrosis and emphysema

**DOI:** 10.1002/cam4.858

**Published:** 2016-08-21

**Authors:** Meng Zhang, Akihiko Yoshizawa, Satoshi Kawakami, Shiho Asaka, Hiroshi Yamamoto, Masanori Yasuo, Hiroyuki Agatsuma, Masayuki Toishi, Takayuki Shiina, Kazuo Yoshida, Takayuki Honda, Ken‐ichi Ito

**Affiliations:** ^1^ Division of BreastEndocrine and Respiratory Surgery, Department of Surgery (II)Shinshu University School of MedicineMatsumotoJapan; ^2^ Department of Laboratory MedicineShinshu University HospitalMatsumotoJapan; ^3^ Department of Diagnostic PathologyKyoto University HospitalKyotoJapan; ^4^ Department of RadiologyShinshu University HospitalMatsumotoJapan; ^5^ First Department of Internal MedicineShinshu University HospitalMatsumotoJapan; ^6^ Department of Thoracic SurgerySuwa Red Cross HospitalSuwaJapan

**Keywords:** Combined pulmonary fibrosis and emphysema (CPFE), histology, lung cancer, prognosis, pulmonary fibrosis

## Abstract

Combined pulmonary fibrosis and emphysema (CPFE) is an important risk factor for lung cancer (LC), because most patients with CPFE are smokers. However, the histological characteristics of LC in patients with CPFE (LC‐CPFE) remain unclear. We conducted this study to explore the clinicopathological characteristics of LC‐CPFE. We retrospectively reviewed data from 985 patients who underwent resection for primary LC, and compared the clinicopathological characteristics of patients with LC‐CPFE and non‐CPFE LC. We identified 72 cases of LC‐CPFE, which were significantly associated with squamous cell carcinoma (SqCC) histology (*n* = 46, *P* < 0.001) and higher tumor grade (*n* = 44, *P* < 0.001), compared to non‐CPFE LC. Most LC‐CPFE lesions were contiguous with fibrotic areas around the tumor (*n* = 59, 81.9%), and this association was independent of tumor location. Furthermore, dysplastic epithelium was identified in the fibrotic area for 31 (52.5%) LC‐CPFE lesions. Moreover, compared to patients with pulmonary fibrosis alone in the non‐CPFE group (*n* = 31), patients with CPFE were predominantly male (*P* = 0.008) and smokers (*P* < 0.001), with LC‐CPFE predominantly exhibiting SqCC histology (*P* = 0.010) and being contiguous with the tumor‐associated fibrotic areas (*P* < 0.001). Multivariate analysis revealed that CPFE was an independent predictor of overall survival (hazard ratio: 1.734; 95% confidence interval: 1.060–2.791; *P* = 0.028). Our results indicate that LC‐CPFE has a distinct histological phenotype, can arise from the dysplastic epithelium in the fibrotic area around the tumor, and is associated with poor survival outcomes.

## Introduction

Combined pulmonary fibrosis and emphysema (CPFE) is a clinical syndrome that is characterized by the following findings: a history of smoking; the presence of dyspnea, pulmonary hypertension, and hypoxemia; relatively normal spirometry and lung volumes in the context of severely impaired gas exchange; radiographically confirmed upper‐lobe emphysema; lower‐lobe fibrosis; and poor survival 1, 2, 3, 4, 5. Since Cottin et al. reported their comprehensive study of patients with CPFE in 2005, CPFE has gradually gained the attention of researchers, and is now recognized throughout the world 2, 4. As almost all patients with CPFE are smokers 2, 4, CPFE has been recognized as an important risk factor for developing lung cancer 4, 6, 7, 8, 9, 10. In addition, lung cancers in patients with CPFE exhibit a high rate of squamous cell carcinoma histology, advanced pathological staging, and relatively short survival 7, 9, 10, 11, 12, 13, 14. Therefore, it is important to clarify the clinicopathological features of lung cancer in patients with CPFE, in order to understand the pathogenesis of CPFE‐related lung cancer and to develop appropriate treatments. However, the association of lung cancer histology with the background histological changes in patients with CPFE remains unclear. Therefore, we conducted this retrospective study to evaluate the histological characteristics of lung cancer in patients with CPFE, using their resected lung specimens.

## Materials and Methods

We retrospectively evaluated cases of lung resection for pulmonary masses, which were treated at Shinshu University Hospital between December 1995 and December 2013, using the hospital's Thoracic Surgery and Pathology database. We used the patients' electronic medical records to retrieve their clinical data, which included age, sex, smoking status, tumor location (including laterality and lobe), history of connective tissue disease and drug treatment, and follow‐up data. Our research protocol was reviewed and approved by our institutional ethics review committee.

**Figure 1:**
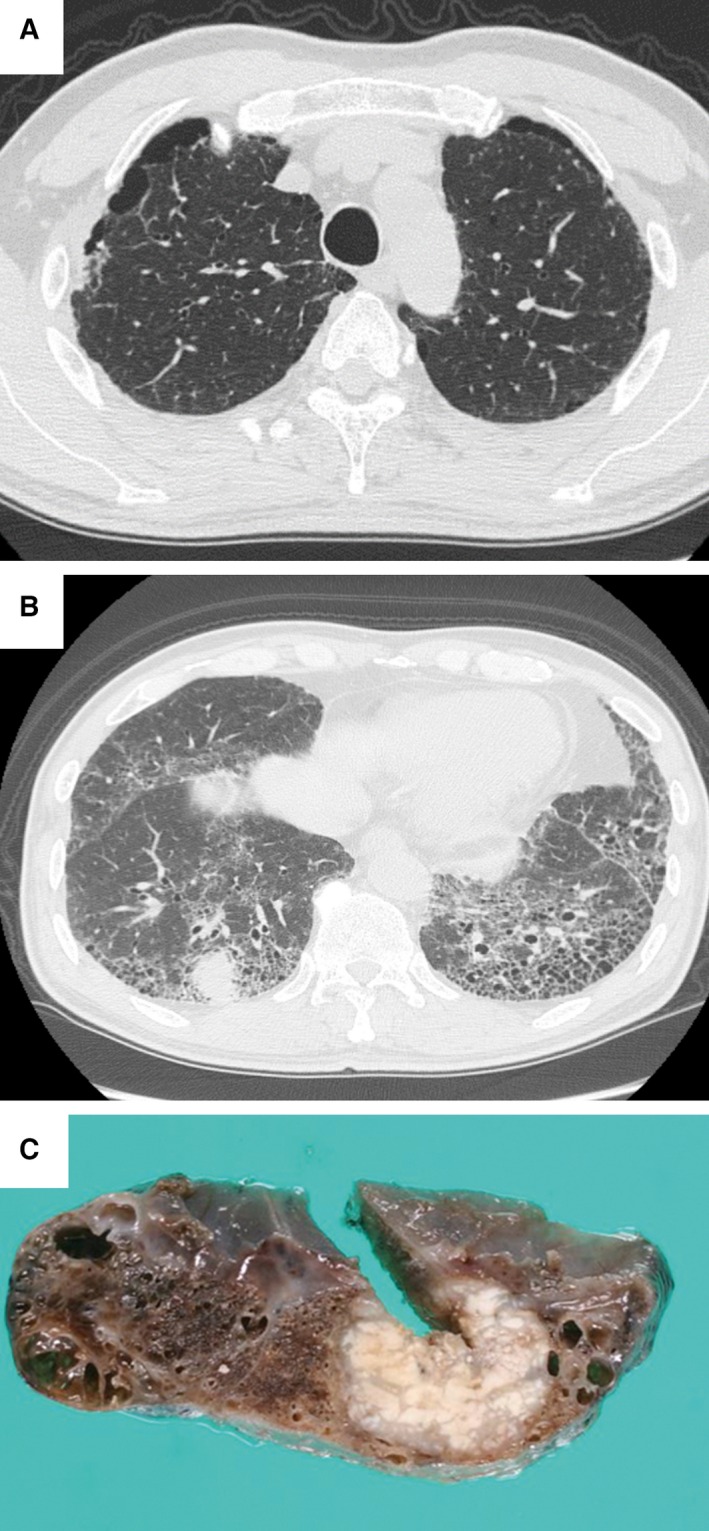
A representative case of lung cancer in a patient with combined pulmonary fibrosis and emphysema**.** Computed tomography reveals (A) bilateral emphysematous changes in the upper lobes and (B) a solid mass within the subpleural fibrous‐reticular area in the right lower lobe. (C) The resected lung specimen reveals a white‐tan tumor along the fibro‐cystic area.

To evaluate the clinicopathological significance of lung cancer in patients with CPFE (LC‐CPFE) after complete tumor resection, we divided patients into LC‐CPFE and non‐CPFE lung cancer groups based on their chest high‐resolution computed tomography (HRCT) findings 7. Lungs with CPFE were diagnosed using Cottin et al.'s criteria 2: (1) the presence of emphysema on CT scan, which was defined as well‐demarcated areas of decreased attenuation in comparison with contiguous normal lung and marginated by a very thin wall (<1 mm), no wall, and/or multiple bullae (>1 cm) with upper zone predominance; and (2) the presence of diffuse parenchymal lung disease with significant pulmonary fibrosis on CT scan, which was defined as reticular opacities with peripheral and basal predominance, honeycombing, architectural distortion, and/or traction bronchiectasis or bronchiolectasis. Focal ground‐glass opacities and/or areas of alveolar condensation are permissible, although these areas should not be prominent (Fig. 1).

We also divided the non‐CPFE group into three groups to evaluate the prognostic significance of LC‐CPFE based on their HRCT images (99.4% of the cases were evaluated using HRCT): lung cancer in patients with only pulmonary fibrosis (LC‐PF), patients with only emphysema (LC‐Emp), and patients with normal lungs (LC‐Norm). The radiographical criteria for CPFE were used to define the LC‐Emp group (fulfilled criterion 1 only), the LC‐PF group (fulfilled criterion two only), and the LC‐Norm group (fulfilled neither criteria). To account for interobserver variation, we omitted the LC‐Norm group, and the LC‐CPFE, LC‐PF, and LC‐Emp groups were independently determined by MZ, AY, a radiologist (SK), and two pulmonologists (MY, HY). All reviewers were blinded to the patients' information, and consensus was used to resolve discrepancies in the group assignments. At this point, we also excluded patients with pulmonary fibrosis that was induced by connective tissue disease and drug treatment from the LC‐CPFE and LC‐PF groups, based on their medical records.

**Figure 2:**
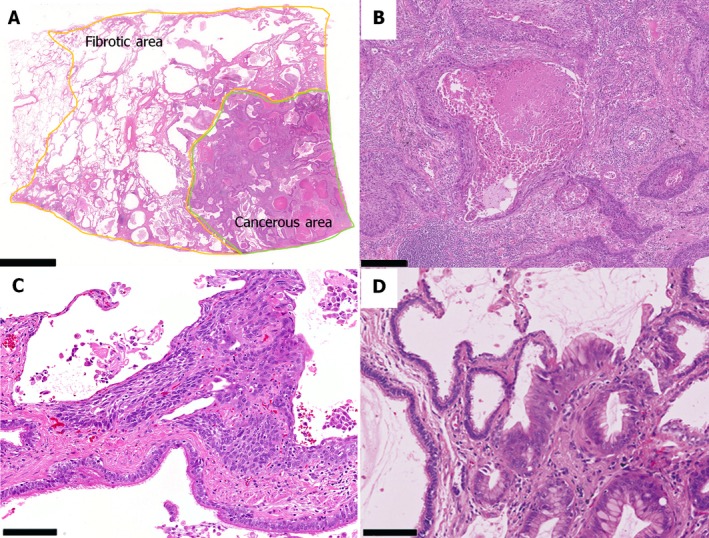
Representative histological features of lung cancer in a patient with combined pulmonary fibrosis and emphysema**.** (A) A low‐power view of lung cancer from a patient with combined pulmonary fibrosis and emphysema (hematoxylin and eosin staining). The cancerous area is enclosed by the green line, and the fibrotic area around the tumor is enclosed by the orange line. (B) A high‐power view of the cancerous area from (A), which reveals invasive squamous cell carcinoma. (C) A high‐power view of the fibrotic area from (A), which reveals the dysplastic squamous epithelium. (D) A high‐power view of the surrounding fibrotic area from another case, which reveals the dysplastic glandular epithelium next to the metaplastic bronchial epithelium. Bars indicate 5 mm for (A), 250 *μ*m for (B), 100 *μ*m for (C), and 100 *μ*m for (D).

To explore the histological characteristics of LC‐CPFE, all histological slides from that group were reviewed under a multiheaded microscope by MZ and two pathologists (AY, SA), who were blinded to the clinical and radiological findings. In addition, the histological slides from the LC‐PF group were reviewed in a similar manner, and their clinicopathological characteristics were compared to those of the LC‐CPFE group. The histological subtype was determined according to the 2015 WHO classification for lung tumors 15, and the TNM stage was determined according to the 7th edition of the Tumor, Node, and Metastasis classification of the International Union Against Cancer 16. Furthermore, we histologically evaluated the area surrounding the tumor for various changes, including the presence or absence of emphysema (with coverage around the tumor in 5–10% increments) and the presence or absence of pulmonary fibrosis (with coverage around the tumor in 5–10% increments). If a fibrotic area was present around the tumor, we also recorded the absence or presence (and percent coverage) of metaplastic epithelium, atypical metaplastic epithelium, or dysplastic epithelium (both squamous and glandular) within the fibrotic area (Fig. 2). To distinguish direct extension of the lung cancer into the fibrotic area from isolated dysplastic epithelium, we defined dysplastic epithelium as foci that were clearly separated from the border between the cancerous and fibrotic areas and that were accompanied by metaplastic epithelium 17.

### Statistics

The chi‐square test and Fisher's exact test were used for categorical data, as appropriate. Mann–Whitney U tests were used for continuous data. The survival rates were calculated using the Kaplan–Meier method, and were compared using the log‐rank test. Multivariate analysis was performed using Cox's proportional hazards model. All statistical tests were two‐sided and used a 5% level of significance. All data analyses and graphing were performed using JMP software (version 8; SAS Institute, Cary, NC).

## Results

### Comparing the clinicopathological characteristics of LC‐CPFE and the other groups

This study evaluated records from 1647 patients; patients without CT images or sufficient clinical and histological information, patients with metastatic tumors, and patients who had undergone chemo‐radiotherapy before surgery were excluded. Therefore, we included 985 patients in this study, and discovered that this group included 72 patients with LC‐CPFE, 82 patients with LC‐Emp, 31 patients with LC‐PF, and 800 patients in the LC‐Norm group. The clinicopathological characteristics of the patients with LC‐CPFE and non‐CPFE lung cancer are summarized in Table 1. Patients with LC‐CPFE were all smokers, with a mean Brinkman index of 1131.7 ± 490.8. Not all patients with LC‐CPFE underwent pulmonary function testing (available data for the CPFE group: FVC [% of predicted], 104.8 ± 19.7%; FEV1.0 [% of predicted], 103.2 ± 22.2%; D_LCO_, 50.9 ± 16.5%), and there were no patients with CPFE and pulmonary hypertension, as patients with pulmonary hypertension are excluded from lung cancer resection at our institution. Compared to patients with non‐CPFE lung cancer, patients with LC‐CPFE were predominantly men (*n* = 67, 93.0%, *P* < 0.001) and older (mean age: 70.5 ± 7.3 years, *P* = 0.012). Furthermore, LC‐CPFE was associated with a larger tumor size (mean size: 29.5 ± 16.0 mm, *P* < 0.001). The numbers of LC‐CPFE cases at each pathological stage were 20 (27.7%) at stage IA, 22 (30.5%) at stage IB, 14 (19.4%) at stage IIA, 2 (2.7%) at stage IIB, 10 (13.9%) at stage IIIA, and 4 (5.6%) at stage IV; LC‐CPFE was associated with a significantly higher stage, compared to the other groups (*P* < 0.001). The most common histological subtype of LC‐CPFE was squamous cell carcinoma (*n* = 46, 63.8%), which was followed by adenocarcinoma (*n* = 19, 26.3%), large cell carcinoma (*n* = 5, 6.7%), small cell carcinoma (*n* = 1, 1.4%), and large cell neuroendocrine carcinoma (*n* = 1, 1.4%). The tumor grade of the LC‐CPFE group was significantly higher than those in the other groups (*P* < 0.001).

**Table 1:** The clinicopathological characteristics of lung cancer in patients with combined pulmonary fibrosis and emphysema (CPFE) and non‐CPFE conditions

Parameter		Total	LC‐CPFE	LC‐non‐CPFE	*P*‐value
		985	72	913	
Age	(mean)	67.5 ± 9.4	70.5 ± 7.3	67.2 ± 9.5	0.012
Sex	Male	540	67	473	<0.001
	Female	445	5	440	
Smoking his.	Current/Former	501	72	429	<0.001
	Never	466	0	466	
BI	(mean)	508 ± 713	1131.7 ± 490.8	458.3 ± 705.3	<0.001
Tumor size	(mean, mm)	23.1 ± 14.9	29.5 ± 16.0	22.6 ± 14.7	<0.001
Stage	IA	601	20	581	<0.001
	IB	194	22	172	
	IIA	73	14	59	
	IIB	48	2	46	
	IIIA	50	10	40	
	IIIB	9	0	9	
	IV	10	4	6	
His. subtype	ADC	779	19	760	<0.001
	SqCC	152	46	106	
	SmCC	6	1	5	
	LCNEC	18	1	17	
	LCC	13	5	8	
	ADSQ	9	0	7	
	Others	8	0	8	
Tumor Grade	G1	411	0	411	<0.001
	G2	320	28	292	
	G3‐4	254	44	210	

Smoking his., smoking history; BI, Brinkman index; His. Subtype, Histological subtype; ADC, adenocarcinoma; SqCC, squamous cell carcinoma; SmCC, small cell carcinoma; LCNEC, large cell neuroendocrine carcinoma; LCC, large cell carcinoma; ADSQ, adenosquamous carcinoma.

### Survival analysis

**Figure 3:**
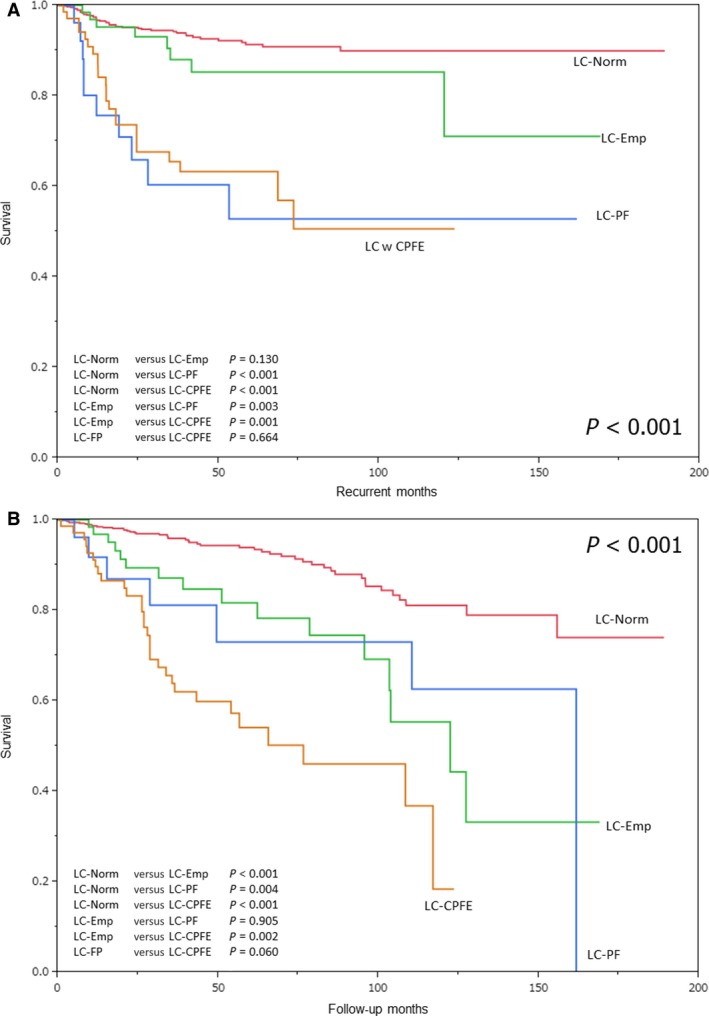
Survival curves. (A) Disease‐free survival and (B) overall survival. LC‐Norm: lung carcinoma in the normal lung, LC‐Emp: lung carcinoma in patients with emphysema, LC‐PF: lung carcinoma in patients with pulmonary fibrosis, LC‐CPFE: lung carcinoma in patients with combined pulmonary fibrosis and emphysema.

The curves for overall survival (OS) and disease‐free survival (DFS) in the four groups are shown in Figure 3. The LC‐CPFE group exhibited a significantly poorer DFS, compared to the LC‐Norm (*P* < 0.001) and LC‐Emp (*P* = 0.001) groups (Fig. 3A). However, patients with LC‐CPFE and LC‐PF had similar DFS (*P* = 0.664). The LC‐CPFE group exhibited significantly poorer OS compared to the LC‐Norm (*P* < 0.001) and LC‐Emp (*P* = 0.002) groups (Fig. 3B). Similarly, the LC‐CPFE group exhibited a poorer OS compared to the LC‐PF group, although the difference was not statistically significant (*P* = 0.060). Patients with CPFE adenocarcinoma or squamous cell carcinoma exhibited poorer survival outcomes compared to patients with non‐CPFE adenocarcinoma (OS, *P* < 0.001; DFS, *P* < 0.001) or non‐CPFE squamous cell carcinoma (OS, *P* < 0.001; DFS, *P* = 0.007).

Table 2 shows the results for the univariate and multivariate analyses of the various clinicopathological factors that we examined. The univariate analysis revealed numerous significant risk factors for poor DFS and OS, and patients with CPFE exhibited a higher risk of recurrence (hazard ratio [HR]: 4.641; 95% confidence interval [CI]: 2.824–7.358; *P* < 0.001) and mortality (HR: 4.993; 95% CI: 3.195–7.595; *P* < 0.001) compared to the patients without CPFE. The multivariate analysis using the Cox proportional hazards model revealed that CPFE was a significant and independent predictor of OS (HR: 1.734; 95% CI: 1.060–2.791; *P* = 0.028), although it was not an independent predictor of DFS (HR: 1.689; 95% CI: 0.974–2.873; *P* = 0.061).

**Table 2:** Univariate and multivariate analyses of the clinicopathological parameters

Parameter	DFS Univariate			DFS Multivariate		
	HR	95%CI	*P*‐value	HR	95%CI	*P*‐value
Age(≥70 vs. <70)	1.176	0.768–1.791	0.452	1.063	0.686–1.638	0.781
Sex(male vs. female)	2.401	1.523–3.912	<0.001	0.966	0.472–2.052	0.928
Smoking status(ever vs. never)	3.099	1.942–5.138	<0.001	1.289	0.565–2.948	0.548
Stage(II‐IV vs. I)	4.236	2.775–6.440	<0.001	2.486	1.558–3.960	<0.001
Histology(non‐ADC vs. ADC)	4.904	3.227–7.483	< 0.001	2.602	1.543–4.462	<0.001
Tumor grade(G3‐4 vs. G1‐2)	3.304	2.167–5.024	<0.001	1.452	0.900–2.347	0.125
CPFE status(CPFE vs. non‐CPFE)	4.641	2.824–7.358	<0.001	1.689	0.974–2.873	0.061

Parameter	OS Univariate			OS Multivariate		
	HR	95%CI	*P*‐value	HR	95% CI	*P*‐value
Age(≥70 vs. <70)	2.636	1.776–3.945	<0.001	2.277	1.520–3.438	<0.001
Sex(male vs. female)	5.087	3.078–8.983	<0.001	1.699	0.784–3.816	0.183
Smoking status(ever vs. never)	5.649	3.447–9.835	<0.001	1.917	0.863–4.406	0.111
Stage(II‐IV vs. I)	2.883	1.929–4.254	<0.001	1.581	1.007–2.459	0.046
Histology(non‐ADC vs. ADC)	4.671	3.190–6.864	<0.001	1.942	1.244–3.068	0.003
Tumor grade(G3‐4 vs. G1‐2)	3.078	2.089–4.513	<0.001	1.336	0.852–2.091	0.205
CPFE status(CPFE vs. non‐CPFE)	4.993	3.195–7.595	<0.001	1.734	1.060–2.791	0.028

DFS, disease‐free survival; OS, overall survival; HR, hazard ratio; CI, confidence interval; ADC, adenocarcinoma.

### Histological characteristics of LC‐CPFE

Table 3 shows the associations of the histological change in the tumor background with tumor location and histological subtype. Among the 72 cases of LC‐CPFE, tumors were most common in the right lower lobe (*n* = 28, 38.8%), which was followed by the right upper lobe (*n* = 19, 26.3%), the left lower lobe (*n* = 12, 16.6%), the left upper lobe (*n* = 11, 15.2%), and the right middle lobe (*n* = 2, 2.7%). Approximately half of the LC‐CPFEs were located in the lower lobes (*n* = 40, 55.5%). Most cases of LC‐CPFE exhibited fibrotic changes around the tumor (*n* = 59, 81.9%), although seven cases of LC‐CPFE exhibited emphysematous changes around the tumor, and six cases of LC‐CPFE did not exhibit fibrotic or emphysematous changes around the tumor. We observed metaplastic epithelium in the fibrotic area around the tumor in all cases (involved area: 5–100%, mean: 45.3%), atypical metaplastic epithelium in 33 cases (involved area: 5–30%, mean: 8.3%), and dysplastic epithelium in 31 cases (involved area: 5–20%, mean: 45.3%). Most cases with the squamous cell carcinoma subtype were located contiguous with the fibrotic area (*n* = 39, 84.7%), and dysplastic squamous epithelium in the fibrotic area was identified in approximately half of these cases (*n* = 20, 51.2%). Most cases with the adenocarcinoma subtype were also located contiguous with the fibrotic area (*n* = 15, 78.9%), and dysplastic glandular epithelium in the fibrotic area was identified in more than half of these cases (*n* = 10, 66.6%).

**Table 3:** The associations of histological changes in the tumor background with tumor location and histological subtype

Histological change around the tumor	Tumor Location					Histological subtype					Total
	RUL	RML	RLL	LUL	LLL	SqCC	ADC	LCC	SmCC	LCNEC	
Normal	0	0	1	4	1	4	2	0	0	0	6
Emp.	5	1	1	0	0	3	2	2	0	0	7
Fibrotic ()^a^	14 (4)	1 (0)	26 (14)	7 (5)	11 (8)	39 (20)	15 (10)	3 (1)	1 (0)	1 (0)	59 (31)
Total	19 (4)	2 (0)	28 (14)	11 (5)	12 (8)	46 (20)	19 (10)	5 (1)	1 (0)	1 (0)	72 (31)

Emp., Emphysematous change; Fibrotic., fibrotic change; RUL, right upper lobe; RML, right middle lobe; RLL, right lower lobe; LUL, left lower lobe; LLL, left lower lobe; SqCC, squamous cell carcinoma; ADC, adenocarcinoma; LCC, large cell carcinoma; SmCC, small cell carcinoma; LCNEC, large cell neuroendocrine carcinoma.

a The number of () indicates the case with dysplastic epithelium in the fibrotic area.

Among the adenocarcinoma cases in the LC‐CPFE group (*n* = 19), the most common subclass was solid adenocarcinoma (*n* = 7, 36.8%), which was followed by papillary adenocarcinoma (*n* = 5, 26.3%), acinar adenocarcinoma (*n* = 4, 21.0%), invasive mucinous adenocarcinoma (*n* = 2, 10.5%), and lepidic adenocarcinoma (*n* = 1, 5.2%) (Table 4). There were no cases of adenocarcinoma in situ or minimally invasive adenocarcinoma, and no lepidic components were observed in the majority of the cases (*n* = 12, 63.1%).

**Table 4:** The clinicopathological characteristics of lung cancer in patients with combined pulmonary fibrosis and emphysema (LC‐CPFE) or pulmonary fibrosis (LC‐PF)

Parameter		Total	LC‐CPFE	LC‐PF	*P*‐value
		103	72	31	
Age		70.4 ± 7.1	70.3 ± 7.3	71.3 ± 7.3	0.751
Sex	Male	90	67	23	0.008
	Female	13	5	8	
Smoking his.	Current/Former	92	72	20	<0.001
	Never	11	0	11	
BI	(mean)	978.2 ± 562.0	1131.7 ± 490.8	622.8 ± 97.4	<0.001
Tumor size	(mean, mm)	32.5 ± 20.8	29.5 ± 16.0	39.6 ± 28.2	0.036
Stage	I	60	42	18	0.380
	II	23	16	7	
	III	15	10	5	
	IV	5	4	1	
Acute Exa.	Positive	8	5	3	0.634
	Negative	95	67	28	
Tumor location	RUL	29	19	10	0.999
	RML	4	2	2	
	RLL	37	28	9	
	LUL	16	11	5	
	LLL	17	12	5	
His. Subtype	ADC	36	19	17	0.010
	SqCC	55	46	9	
	SmCC	2	1	1	
	LCNEC	3	1	2	
	LCC	7	5	2	
Tumor grade	G1	6	0	6	0.441
	G2	39	28	11	
	G3‐4	58	44	14	
When ADC, subclassification	Lepidic	3	1	2	0.639
	Acinar	10	4	6	
	Papillary	11	5	6	
	Micropapillary	2	0	2	
	IMA	2	2	0	
	Solid	8	7	1	
When ADC, lepidic comp.	Presence	15	7	8	0.534
	Absence	21	12	9	
Histological change around the tumor	Normal	27	6	21	<0.001
	Emp.	7	7	0	
	Fibrotic ()^a^	69 (33)	59 (31)	10 (3)	

Smoking his., smoking history; BI, Brinkman index; Acute Exa., acute exacerbation; His. Subtype, histological subtype; lepidic comp., lepidic component; RUL, right upper lobe; RML, right middle lobe; RLL, right lower lobe; LUL, left lower lobe; LLL, left lower lobe; ADC, adenocarcinoma; SqCC, squamous cell carcinoma; SmCC, small cell carcinoma; LCNEC, large cell neuroendocrine carcinoma; LCC, large cell carcinoma; IMA, invasive mucinous adenocarcinoma; Emp., Emphysematous change; Fibrotic, fibrotic change.

a The number of () indicates the case with dysplastic epithelium in the fibrotic area.

### Comparing LC‐CPFE and LC‐PF

The clinicopathological characteristics of the patients with LC‐CPFE and LC‐PF group are summarized in Table 4. Compared to the LC‐PF group, patients with LC‐CPFE were predominantly men (67 vs. 23, *P* = 0.008) and smokers (72 vs. 20, *P* < 0.001). Furthermore, LC‐CPFE was associated with a significantly smaller tumor size (mean: 29.5 mm vs. 39.6 mm, *P* = 0.036). The most common histological subtype of LC‐CPFE was squamous cell carcinoma (*n* = 46, 63.8%), and the most common subtype of LC‐PF was adenocarcinoma (*n* = 17, 54.8%). Among the adenocarcinoma cases in the LC‐CPFE group, the most common subclass was solid adenocarcinoma (*n* = 7, 36.8%), and only one case (3.2%) of solid adenocarcinoma was observed in the LC‐PF group. Compared to the LC‐PF lesions, the LC‐CPFE lesions were significantly more likely to be located along the fibrotic area (*P* < 0.001). The lung cancers along the fibrotic area in both groups exhibited dysplastic epithelium in the surrounding fibrotic area, although there was no significant difference between the two groups (*P* = 0.187). Five patients in the LC‐CPFE group experienced acute exacerbation and subsequently died, while three patients died because of acute exacerbation in the LC‐PF group; this difference was not statistically significant (*P* = 0.634).

## Discussion

Since the concept of CPFE was proposed by Cottin et al. 2, various researchers have explored the clinicopathological association between lung cancer and CPFE 7, 9, 10, 12, 13, 14. In this study, we discovered that patients with lung cancer and CPFE were typically smokers, and were predominantly male and older (vs. patients with non‐CPFE lung cancer). Moreover, the lung cancers in this study were predominantly squamous cell carcinoma with high‐grade dysplasia, had progressed to a relatively high stage at the time of diagnosis, and exhibited poor survival outcomes. These findings are generally compatible with those of the previous studies 7, 9, 10, 13, 14. However, those studies generally evaluated the clinical risk and/or incidence of lung cancer in patients with CPFE, and no studies have examined the histological characteristics of lung cancer in patients with CPFE, or the relationship between lung cancer and the histological changes around the tumor. This study demonstrated that lung cancers in patients with CPFE had developed in heterogeneous tumorigenic backgrounds (normal, emphysematous, and fibrotic areas), and that most lung cancers were associated with the fibrotic area around the tumor. Moreover, dysplastic epithelium in the fibrotic area was frequently identified in these cases, which indicates that the development of lung cancer in patients with CPFE is distinct from the development of lung cancer in patients without CPFE.

We found that 55.5% of the lung cancers in patients with CPFE were located in the lower lobes, which is compatible with the findings of the previous studies^9^, ^13^. However, we also found that most of the lung cancers (*n* = 59, 81.9%) were associated with the fibrotic area around the tumor in the patients with CPFE, and this relationship was independent of the tumor's location. Furthermore, a majority of our cases (*n* = 31, 52.5%) exhibited dysplastic epithelium in the fibrotic area. Thus, we hypothesize that pulmonary fibrosis is strongly associated with lung cancer development in patients with CPFE. To the best of our knowledge, this is the first report regarding this relationship in patients with CPFE, although a few researchers have reported similar findings for lung cancer in patients with pulmonary fibrosis. For example, Kawasaki et al. analyzed the relationship between cancer location and regions of idiopathic pulmonary fibrosis (IPF), and reported that 50% of the cancers occurred in the fibrotic parenchyma and that 29% of the cancers occurred in the marginal area of the fibrosis 18. Furthermore, Khan et al. have reported a striking transition from metaplastic squamous epithelium (within the fibrotic areas) to invasive carcinoma in 50% of the resected squamous cell tumors from patients with IPF 17. Based on these findings, it is possible that lung cancer in patients with CPFE has a similar developmental process to that of lung cancer in patients with IPF, as these two groups exhibit increasing epithelial atypia, atypical metaplasia to carcinoma in situ, and invasive carcinoma in the surrounding fibrotic area. However, this possibility is not surprising, as CPFE is not a distinct entity, but rather a state of coexistence between pulmonary fibrosis and emphysema 2, 19. These issues raise the question of whether we should clinically differentiate between LC‐CPFE and LC‐PF. Although most previous studies have highlighted a risk of developing lung cancer in patients with CPFE, and compared their overall prognosis to patients with emphysema or IPF 6, 7, 9, 10, only one study has compared the prognoses of LC‐CPFE and LC‐PF. In that study, Kumagai et al. retrospectively analyzed the effect of CPFE on the prognosis of patients with NSCLC after complete tumor resection 11, and concluded that CPFE was a significant predictor of a poor prognosis for NSCLC. In this study, we found that patients with LC‐CPFE exhibited poorer OS, compared to patients with LC‐PF (although this difference was not statistically significant), despite both groups exhibiting similar DFS. These findings indicate that both groups have a similar risk of recurrence, although different prognoses in terms of OS. We presume that this difference may be related to a reduced respiratory capacity in the lungs of patients with CPFE (because of emphysema that is caused by smoking), compared to the respiratory capacity of patients with IPF. Moreover, our multivariate analyses revealed that CPFE was an independent prognostic factor for OS. Therefore, we believe that CPFE should be differentiated from IPF among patients who have undergone lung resection for lung cancer.

Nineteen of the 72 LC‐CPFE cases exhibited adenocarcinoma histology, with solid adenocarcinoma (*n* = 7, 36.8%) as the most frequent subclass, no cases of adenocarcinoma in situ or minimally invasive adenocarcinoma, and only one case of lepidic adenocarcinoma. Moreover, tumors without a lepidic component were observed in a majority of the cases (*n* = 12, 63.1%). These findings may indicate that lung adenocarcinomas in the CPFE group were different from those in the non‐CPFE group. After the IASLC/ATS/ERS multidisciplinary classifications of lung adenocarcinoma were published in 2011 20, many studies have reported the frequency of lung adenocarcinoma subclasses, with the most common being papillary predominant adenocarcinoma or acinar predominant adenocarcinoma 21, 22, 23, 24, 25. Moreover, no studies have reported solid adenocarcinoma as the most common histological subclass of lung adenocarcinoma. Although we did not histologically compare the lung adenocarcinoma subclasses between patients with and without CPFE in this cohort, we speculate that lung adenocarcinoma in patients with CPFE is a unique lung cancer, which can develop from the dysplastic epithelium in the fibrotic area via a process that is similar to that for squamous cell carcinoma. Furthermore, we observed that most adenocarcinomas in the CPFE group grew along the irregular fibrous wall and intermingled with non‐neoplastic glands in the fibrotic area. In these cases, it is very hard to determine the lung adenocarcinoma subclass, and we question whether these should be considered as the lepidic or acinar pattern. Given all these findings, we suggest that lung adenocarcinoma in patients with CPFE should be considered as a particular subclass (e.g., “invasive adenocarcinoma with fibrosis”). However, further studies with a larger sample size are needed to validate these findings.

In conclusion, the findings of this study indicate that LC‐CPFE may arise from dysplastic epithelium in the fibrotic area around the tumor, and that the process of developing lung cancer may be similar to that of LC‐PF. However, as our patients with LC‐CPFE exhibited significantly poorer outcomes, we suggest that CPFE should be considered as an important background disease for patients who have undergone resection for lung cancer.

## Conflict of Interest

There are no conflicts of interest to declare.
